# ShareDNA: a smartphone app to facilitate family communication of genetic results

**DOI:** 10.1186/s12920-020-00864-0

**Published:** 2021-01-06

**Authors:** Chethan Jujjavarapu, Jeevan Anandasakaran, Laura M. Amendola, Cameron Haas, Elizabeth Zampino, Nora B. Henrikson, Gail P. Jarvik, Sean D. Mooney

**Affiliations:** 1 Department of Biomedical Informatics and Medical Education, School of Medicine, University of Washington, Seattle, WA USA; 2 Institute of Translational Health Sciences, University of Washington, Seattle, WA USA; 3 Department of Medicine (Medical Genetics), University of Washington, Seattle, WA USA; 4 Kaiser Permanente Washington Health Research Institute, Seattle, WA USA; 5 Department of Genome Sciences, University of Washington School of Medicine, Seattle, WA USA

**Keywords:** Genetic testing, Application, Genetics, Cascade testing, Smartphone, App, Family communication

## Abstract

**Background:**

Genetic testing allows patients and clinicians to understand the risk of hereditary diseases. By testing early, individuals can make informed medical decisions about management which may minimize the risk of developing certain diseases. Importantly, genetic test results may also be applicable to patients’ biological relatives; thus, these results could also lead to minimizing their risk of disease. However, sharing genetic test results between patients and their relatives is scarce. The most frequently reported problems are that patients cannot clearly explain this information and relatives misinterpret the results. Smartphone apps in the healthcare field are a possible solution as they allow patients to accurately share sensitive information to others, while providing educational material to support understanding the information. However, these apps may not provide security to protect patients’ identifiable information. We developed *ShareDNA*, a smartphone app that (1) allows patients to securely share their genetic test results with others, (2) provides information on how to interpret these results, and (3) minimizes the amount of patient information needed to use the service.

**Results:**

We recruited thirteen participants to test the usability of our app and provide feedback. We found overall that participants were comfortable with using this app and could easily learn each app function when filling out our questionnaire. Additionally, based on vocalized impressions of the usefulness of the app, participants indicated that the user-interface could be more intuitive and that we needed to add more text within the app to explain why *ShareDNA* is a secure service.

**Conclusions:**

*ShareDNA* is a free smartphone app that allows patients to share their genetic test results with others, including their biological relatives. Sharing these results along with educational material will enable relatives to share accurate information and discuss their possible risk for disease with their clinical providers. As a result, appropriate testing in relatives could be improved.

## Background

Genetic testing is an essential tool to assist patients and clinicians to better understand the risk of hereditary disease. The cost of genetic testing has decreased and the number of genes routinely evaluated has increased in recent years, due to massively parallel sequencing methods and new discoveries [1, 2]. Patients now have increased access to genetic information that can be important for their and their family’s health.

Early genetic testing for germline risk variants can promote reproductive autonomy and lead to the recommendation of appropriate medical screening to mitigate risk of developing disease or provide early diagnosis at a more treatable stage. For example, the most common hereditary disease that elicits a genetic clinic visit and testing in adults is cancer, specifically colorectal cancer (CRC), breast cancer (BC), and ovarian cancer (OC). Approximately 5% of CRC and BC, and 10–20% of OC, is due to high penetrance Mendelian conditions [3–5]. CRC accounts for 9.5% of all new cases of cancer [6]. BC is the second leading cause of cancer death in women; 3% of women in the U.S. will die of BC [7]. OC affects 1–2% of women, most of whom will die from it. To mitigate the cancer-related death rate, early detection of Mendelian (germline) cancer predisposition is of grave importance. If CRC-associated pathogenic variants are found, colonoscopy with polypectomy can prevent CRC and prophylactic bilateral salpingo-oophorectomy surgery reduces OC risk and are consensus recommendations [8, 9]. Similarly, for BC/OC associated genes, prophylactic mastectomy reduces risk of BC [10]. Thus, early genetic testing is necessary to reduce risk of morbidity and mortality for patients.

While genetic testing is important for patients, these test results may also be important for their biological relatives. A patient’s positive test result allows for inexpensive and often free direct testing of at-risk family members for that same pathogenic variant. A positive or negative test in a family member is likely to affect their clinical care. For positive test results, relatives’ treatment plans may change to reduce disease risk, while for negative test results, relatives may not be at increased risk and additional testing may not be necessary [11].

However, sharing of genetic results between patients and their biological relatives is infrequent [11–18]. The two most frequently reported communication barriers for sharing are (1) patients have difficulty in clearly communicating the results and meaning, and/or (2) biological relatives have difficulty in interpreting the result [11–13, 17]. Nieuwenhoff et al. found that patients had limited knowledge of their test results and this influenced whether or not they would share [12]. For example, terms in the test result like “hereditary” implied danger and motivated patients to share, while terms like ‘sensitivity’, ‘tendency’, and ‘it runs in the family’ made patients perceive the results as normal and did not share [12]. Additionally, if patients shared then there was a risk of arousing fear in their relatives, as they couldn’t clearly explain the benefit and risk reduction from getting their own genetic test results [12]. Another recent study reported when patients shared their test results with their biological relatives, over 20% didn’t fully understand the results and were unsure if they were at risk for cancer [11]. This 20% was mainly for non-informative test results, indeterminate results or variants of uncertain significance [11]. As a result, patients’ explanations had a combination of filtering information and lack of understanding [11].

Family communication tools may improve the dissemination of genetic results among family members. With increasing access to mobile phones and devices, mobile technology, such as apps, have become popular methods to share information [19–21]. Studies investigated the value of this technology specifically in families and found that it was a valuable tool for parents and their children to communicate sensitive topics that they didn’t feel comfortable discussing in-person [22]. Additionally, this technology facilitates family members being in contact when they are not geographically close [23]. In the healthcare space, mobile technology provides a means of communication to improve health behavior for patients [20]. However, mobile health apps’ patient privacy is questionable. A recent study found that 81% of diabetes apps do not have privacy policies and would share sensitive patient information to third parties without the patient’s permission [24]. Additionally, another study found that 20% (7/35) of health apps would transmit identifiable information over the Internet without encryption [25]. We believe there is an opportunity to leverage mobile technology to increase communication genetic test results between patients and their family members, while protecting their privacy.

We built *ShareDNA*, a free secure smartphone app, to (1) lower the barrier to sharing genetic test results with family members by sharing test results with anyone contactable by text or email, (2) provide links to educational material and text to explain how to understand the results and proper next steps for recipients, and (3) increase security of patient data by allowing for encrypted transmission and minimizing the amount of data needed to register for the app. Here we describe the development of the *ShareDNA* app, and the results of user testing to inform usability and acceptance.

## Implementation

### Application

#### Overview

**Fig. 1:**
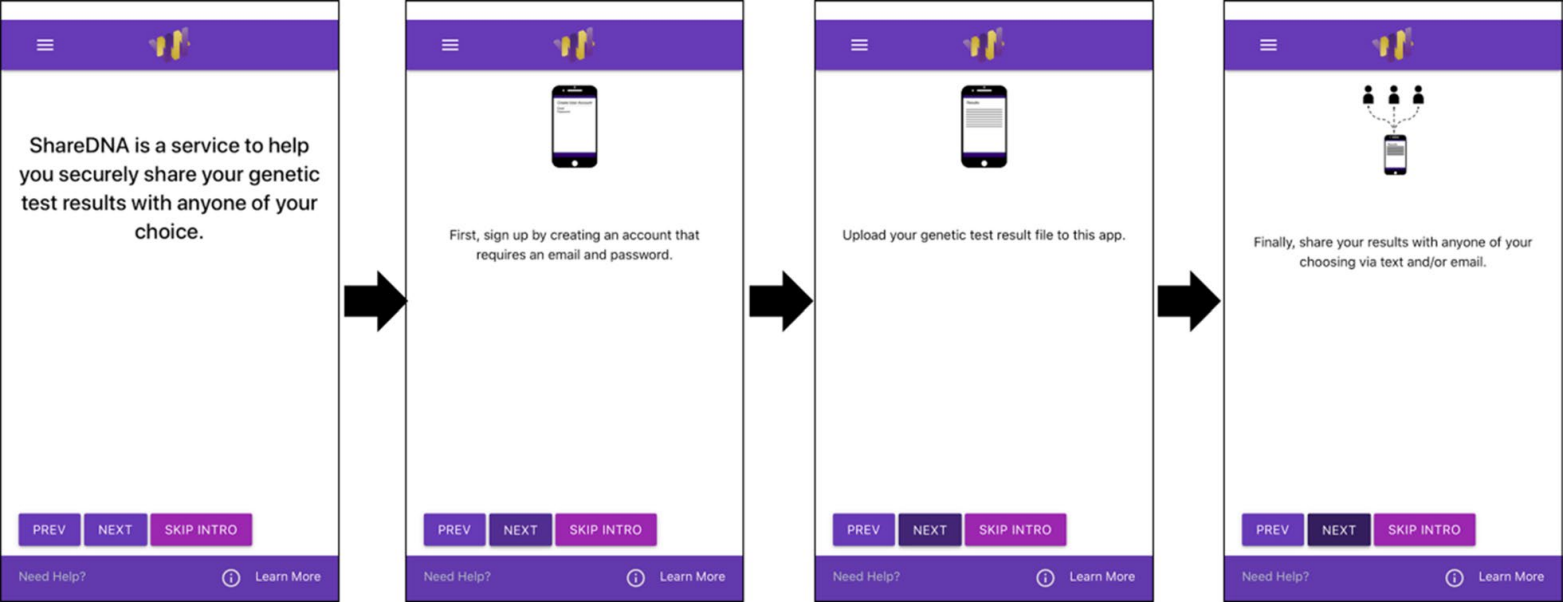
Overview of the purpose of *ShareDNA*. *ShareDNA* provides a service to allow users to create an account that only requires their email and password and then they can upload their genetic test results and share with anyone from their contact list

**Fig. 2:**
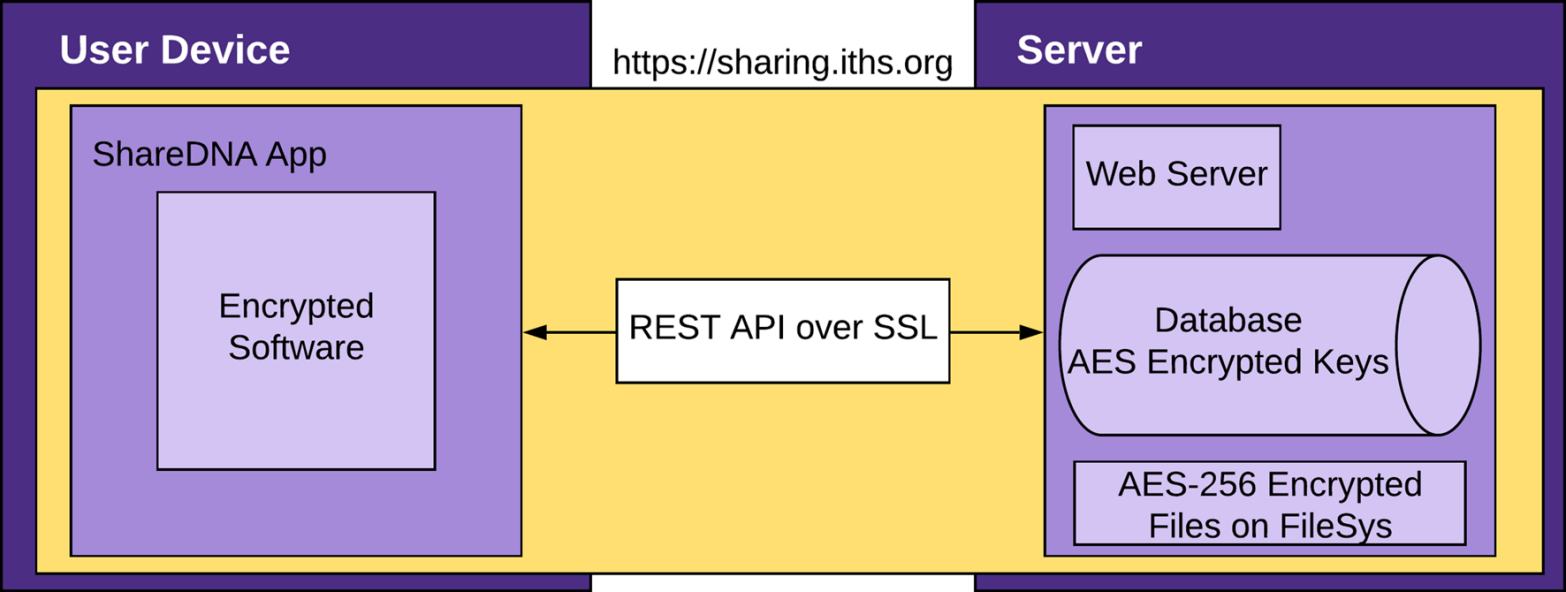
Overview of *ShareDNA’s* communication between the app and server-side. The app side of *ShareDNA* faces the users and when users upload their genetic test results, the information is securely sent to a server maintained by University of Washington for storing

*ShareDNA* is a smartphone application that allows users to share DNA results with family members in a secure way (Fig. 1). The application is divided into two parts: the app and server (Fig. 2).

#### App

The app faces the user and allows them to upload their documents either by selecting a file from their smartphone or using their smartphone’s camera to take a picture of their result. The code itself is encrypted on the user’s smartphone. The app is built using HyperText Markup Language (HTML), JavaScript (JS), and Cascading Style Sheets (CSS). Once uploaded, user’s files are encrypted using ‘AES-256-CBC’ encryption that cannot be decrypted unless a user enters their password again. Users are required to enter their password every time a file is uploaded or downloaded to ensure only the user and their recipients can access the files. All communication from the app to the server is encrypted using Secure Socket Layer (SSL) with a 256-bit Certificate. The app interface was designed using Cordova and is available in the Apple App and Google Play store. With this approach, we came across an obstacle in which Apple app guidelines frequently changed which required refactoring the app, especially before the first production build.

##### Server

User’s encrypted information (i.e. email, password, and test result file) is stored on a Security-Enhanced Linux server with an encrypted file system hosted by University of Washington. The application programming interface (API) is a web application written in Hypertext Preprocessing (PHP) 7 + running on the Apache web server with a MariaDB database for data storage.

### Participants

Participant recruitment was conducted in two phases. We first sent invitations to 49 participants who were enrolled in the Electronic Medical Records and Genomics (eMERGE) network clinical study at Kaiser Permanente Washington who had received positive (pathogenic or likely pathogenic) genetic test results. The eMERGE network is a consortium that develops methods to use electronic health record information for genomic research [26]. In the second phase, we sent a batch of 100 invitations in the mail to eMERGE study participants who had received negative test results. In total, we recruited 14 participants, however one dropped out early in the study for unknown reasons. As a result, we only considered the 13 active participants for this study. For each of the 13 participants, we performed an app testing session. Institute Review Board approval was provided by the Kaiser Permanente Health Research Institute Human Subjects Board. Each participant received a $50 incentive.

### User-testing

To evaluate the usability and acceptance of *ShareDNA*, we used the Technology Acceptance Model (TAM) as a framework [27]. We assessed the perceived ease of use (usability) of the tool through observing the participants walk through the procedures and functions for sending relatives their genetic test results and recorded whether the participant successfully accomplished a list of tasks. Testing of the app was done using an iPad. The app was already installed on the device, as well as a picture of a blank genetic test result and contact information for two fabricated relatives. Each participant was also provided a unique email and password to create an account for the app, along with the contact information for two hypothetical at-risk relatives and a hard copy of blank genetic test results. After being consented, we provided a generic scenario with the task to deliver the test results to the two relatives using the ShareDNA app. We had users fill out a usability testing document that indicated how to perform tasks in the app and allowed for user feedback on what could be improved (Additional file 1). In addition, we used the 16-item version 3 of the Post Study System Usability Questionnaire (PSSUQ), which is a validated instrument designed for usability evaluations, to assess attitudes towards the app after use [28]. The scale is out of five, with one indicating “strongly disagree” and five indicating “strongly agree”. Finally, we asked participants to vocalize their thoughts and impressions while interacting with the app and then recorded their responses. Testing was done in-person.

## Results

### Participants demographics

Our thirteen participants consisted of nine males and four females with an average age of 67.5, minimum age of 60, maximum age of 74, and a standard deviation of 4.8. Our participants were primarily white (10 out of 13).

### User-testing of ShareDNA app

We found on average, the PSSUQ questions with scores above four indicated that users felt comfortable with using this app and could easily learn each app function, however, the lowest scoring question indicated that when users came across a problem, our error messages were not informative enough to help (Table 1). These results indicate that reformatting our error messages is needed to better assist users that may have some difficulties with our app. Additionally, participants vocalized their thoughts about sharing via email. Participants expressed a natural inclination to email, as one participant explained: “because it's just what they've done all their lives.”

**Table 1:** Post-study app usability questionnaire results

Questions	Minimum	Q1	Mean	Median	Q3	Maximum	Standard Deviation
Overall, I am satisfied with how easy it is to use this app	2	3	3.62	4	4	5	0.96
It was simple to use this app	2	3	3.77	4	4	5	0.83
I could effectively complete the tasks and scenarios quickly using this app	2	3	3.92	4	5	5	1.12
I was able to complete the tasks and scenarios quickly using this app	2	2.75	3.5	4	4	5	1.09
I was able to efficiently complete the tasks and scenarios using this app	2	3	3.77	4	4	5	0.83
I felt comfortable using this app	3	3	4.08	4	5	5	0.9
It was easy to learn to use this app	2	4	4	4	5	5	0.91
I believe I could become productive quickly using this app	3	4	4.31	4	5	5	0.63
The app gave error messages that clearly told me how to fix problems	1	2	2.64	2	3	5	1.12
Whenever I made a mistake using the app, I could recover easily and quickly	2	2	3.08	3	4	5	1
The information provided with this app was clear	2	2.75	3	3	3.25	4	0.74
It was easy to find the information I needed	2	3	3.45	4	4	4	0.69
The information provided for the app was easy to understand	2	4	3.83	4	4	5	0.72
The information was effective in helping me complete the tasks and scenarios	3	4	3.92	4	4	5	0.64
The organization of information on the app screens was clear	2	3	3.54	3	4	5	1.05
The interface of this app was pleasant	3	4	3.85	4	4	5	0.55
I liked using the interface of this app	2	3	3.67	4	4	5	0.89
The app has all the functions and capabilities I expect it to have	2	3	3.75	4	5	5	1.14
Overall I am satisfied with this app	2	3	3.77	4	4	5	0.93

Table shows the responses from our cohort of 13 to the PSSUQ. The scale is out of five, with one indicating “strongly disagree” and five indicating “strongly agree”. PSSUQ = Post-Study System Usability Questionnaire

### Issues with app

Users had a number of concerns along with recommendations based on the vocalized impressions of the app (Table 2). We found three main themes: (1) certain aspects of our user interface were not intuitive, such as how to select multiple contacts to send a result to, (2) there was a lack of understanding of our security measures, which is why users were confused as to why they needed to enter their password multiple times, and (3) users were confused with modern icons for buttons, such as *Share* and *Downloading*. The second theme is of particular importance, because there seems to be a lack of understanding of the implications of an information leak.

**Table 2:** Recommended Improvements to *ShareDNA* Issues

Issue	Recommendation
Default messaging was impersonal/generic	Leave blank with suggested wording above the text box. Most participants felt the wording should be in first person since it would come from their number/email
Redundancy in requiring password	Remove additional password requirements once the user is logged into their account. Or an option to require the password before sending test results to recipients
Adding multiple recipients wasn’t intuitive	Add a feature to “save” recipient contact info and the “ + ” to add more recipients
Light greys were difficult to see	Darken grey or change color to indicate the text can be altered
Confusion from intro screens	Once all screens/dialogue has been rotated through (i.e. pressed “Next” 3 times), enter the login/create an account screen automatically
“Create an account” was overlooked	If the email entered does not have an account yet, navigate to the “create an account” page with the information already entered
“Share” icon wasn’t clear	Older users didn’t intuitively know the icon to share and the font was small, a larger button with text would be clearer
Scrolling function wasn’t shown	Add scrolling sidebar to “More information” section to show additional text is below
UW branding was confusing to non-UW patients	Consider de-emphasizing UW look and feel if using with external patients
Some participants didn’t intuitively go to “Files” to send their test results again	From upper left menu, include a “Share” option

Table outlines the major issues that participants found along with their recommended improvements

## Discussion

### User-interface

#### Creating an account and educational material

Once the app is opened, *ShareDNA* describes the purpose of the app with a mission statement along with visual slides (Fig. 1). The user needs to create an account by providing an email address and password to log in to the app. Both the password and email are one-way encrypted, so that they are not stored on the server. Additionally, if the user requires further assistance on how to use the app, they can tap the *Need Help?* Icon on the bottom left of the screen to contact the *ShareDNA* team. Finally, *ShareDNA* provides links to websites that provide educational material. Two of the provided links direct users to our local medical genetics clinic at the University of Washington and to genetic counseling resources across the United States (the National Society of Genetic Counselors 'find a provider' page), particularly for individuals with questions and/or who have a positive result and need follow up care. Additionally, we provide links to two websites with reliable, general genetic condition (Medline) and hereditary cancer specific (National Cancer Institute) information written for the general population for users who may want to research a given condition on their own. The users tap the *Learn More* icon on the bottom right of the screen to access these links.

#### Upload a genetic test result

When a user uploads a file either from their smartphone’s local storage or taking a picture with their smartphone’s camera, the server uses a randomly generated key to encrypt the file and save to the filesystem; the server then erases the key making the file only accessible for sharing and downloading if the user enters their password again.

#### Sharing file through text and/or email

The user can view a list of their uploaded files and select to share a file. The user must enter their password again which allows the server to create message keys that can be later used by the server to decrypt the files sent to the recipient. Once the device receives the temporary message key for the file, they can select to send a message to a single contact or multiple contacts through the smartphone’s native email or text messaging application. Once selected, they can send a message containing a link to the *ShareDNA* web application allowing the recipients to register and access the file. These message keys are only available for 24 h and can only be used once per recipient; this is to avoid leaving them hanging in emails and texts for the wrong people to read and prevent brute force attacks on our server.

#### Recipient viewing the file

After sharing a file, the recipient will receive a link either through email or text. The link takes the recipient to the ShareDNA website, where the recipient will need to create an account and log in to access the file. Once logged in, the recipient can tap the “Testing” button next to the file to learn about the next steps after viewing the file. These steps include (1) how to interpret the results and (2) taking the file to their clinician to discuss if genetic counseling is necessary or not. In order to download a file to their device, recipients must enter their password again. The password is sent to the server to decrypt the file and creates a new temporary file encrypted with a new key that is sent to the device and not stored on the server. The user then uses this key along with their logged in API key to decrypt and download the file to their device. Once the file is on their device, it is stored to a location of their choice in an unencrypted state.

### New features to add

In a future implementation, we will need to address our app testers’ concerns by (1) allowing future users to guide us in implementing the logical steps needed to execute our functions, (2) include documentation in the app that explains why security is needed for clinical data, and (3) using text rather than icons to describe our buttons.

### Comparison to other existing software

*ShareDNA* is similar to the app *FamGenix* [18]. Both apps (1) allow patients to share their genetic information with anyone of their choosing through text or email and (2) store their information on a secure Health Insurance Portability and Accountability Act (HIPAA) compliant server with encryption at rest and in transit. The difference is that *ShareDNA* is a free service focused on sharing genetic test results, while *FamGenix* is a paid sharing service with data analytics. *FamGenix* employs genetic risk algorithms to autogenerate pedigrees and calculate the hereditary cancer risk for a patient; both can be shared with patients’ family members. While useful, we believe sharing algorithm-derived risk scores could lead to (1) misinterpretation as these risk scores should only be considered as aids for diagnosis and/or (2) incorrect results. We believe our approach of encouraging patients’ family members to share their information with their healthcare provider is a safer option as it (1) minimizes the possibility of misunderstanding and (2) emphasizes healthcare providers’ valuable expertise and experience.

## Conclusions

*ShareDNA* is a free secure smartphone app that allows patients to share their genetic test results with others, with emphasis on their family members who may benefit from this information. The main benefit of the app is to provide a secure environment for sharing the genetic test report by requiring minimal user information and encrypting the storage and transportation of data. Our app addresses patients’ difficulty in communication and their relatives’ difficulty in interpretation by providing links to educational websites to learn more about genetic testing and text to explain how to interpret these results and next steps for their relatives to get their own testing if needed. Our user-testing indicated that participants felt comfortable with our app, however improvements were suggested to better support potential users, specifically understanding the importance of our security measures (i.e. entering their password twice). Our next immediate step will be to implement our participants’ recommendations (Table 2). A future step would be to perform another usability test to further explore the TAM framework, specifically usefulness and intention to use, by expanding our questions for participants to include ones that directly ask about the usefulness of the app and its educational material and the intention to use the app. A limitation of our study is the sample size (13 participants). A larger sample size may have provided more feedback on how to improve our app. Another limitation is our results are limited to an age group favoring elderly individuals (atleast 60 years old). Another limitation is that our participants came from the eMERGE consortium only. These individuals are familiar with genetic testing, so they may not represent the general population. As a result, our findings may be limited in their generalizability. To address these three limitations, a future step would be to perform another usability test, as stated before, but with a larger cohort of participants that come from both the eMERGE consortium and general population with a wider age range. A final limitation is that participants had some familiarity with using a smartphone. While a limitation, this is a necessary prerequisite to use our app. We believe *ShareDNA* will become a useful tool to promote timely communication of genetic risk information to ensure family members are able to make informed decisions about whether or not to access genetic testing. Important features enabled by *ShareDNA,* include file sharing, data encryption, and links to resources, could reduce the barriers to successful cascade screening programs.

## Availability and requirements

Project name: ShareDNA, Project home page: http://sharedna.org/*,* Project source code page: https://github.com/uwrit/AppShareDNA*,* Operating system(s): iOS and Android, Programming language: JS, HTML, CSS, PHP, Other requirements: None, License: MIT, Any restrictions to use by non-academics: License required.

## Supplementary Information

**Additional file 1:** File shows the results for each participant walking through and providing feedback for each function in the app.

## Data Availability

The data generated during the current study are not publicly available due to the data containing Protected Health Information but are available from the corresponding author on reasonable request. All data analysed during this study are included in this published article [and its supplementary information files].

## Abbreviations

CRC: Colorectal Cancer
BC: Breast Cancer
OC: Ovarian Cancer
HTML: HyperText Markup Language
JS: JavaScript
CSS: Cascading Style Sheets
SSL: Secure Socket Layer
API: Application Programming Interface
PHP: Hypertext Preprocessing
eMERGE: Electronic Medical Records and Genomics
TAM: Technology Acceptance Model
PSSUQ: Post Study System Usability Questionnaire
HIPAA: Health Insurance Portability and Accountability Act
